# A Home Sister of the Bygones

**Published:** 1917-12-01

**Authors:** 


### SOME HOSPITAL TYPES OF BRITISH NURSING.

### II. A Home Sister of the Bygones.

AND the Home Sister that I dare to attempt to outline was one of bygone days.

Made Home Sister for her rare ability, asked to leave the Ward she loved for this more urgent call, and remaining in her post many years, not resigning it until she thought she was not able to give as much as in earlier days.

Present at the Probationers' Breakfast at half-past six. Reading prayers with an eye for late comers.

The first up, the last to Bed. With Military precision enforcing Punctuality, Cleanliness, and Order.

A Character singularly Sincere, Simple, and Single-minded.

One of the most thorough Workers and Teachers. Her Classes were an Inspiration, for she brought to them a full mastery of the subject—a clear, interested, and interesting presentment.

She had sympathy with the feebler sort, but the wilfully stupid woman received scant attention.

Her Standard of Life and Work was high, and her Influence on the Probationers—most of them still at an impressionable age—was all for good.

She was a Menace to Slackers and Evildoers, and some Ward Sisters were a little Restive at her Sharp Eye—with a feeling that there existed an unspoken sarcastic criticism.

She was rarely seen in the Wards, although fascinated by a pretty child, and she often had the little ones to Tea in her Room in the Home.

We used to think she was afraid of being disappointed in her Nurses if she saw them "On Duty."

She always paid a Sister due Honour and Courtesy, but she expected much from the position, and Gossip, Scandal, and Slang were abhorrent to her straightforward Nature.

Dear Home Sister, apparently stern, and expecting more than many cared or were able to give, thinking the Work should make Saints of us, yet so tender and self-denying if anyone—Probationer, Nurse, or Sister—was sick or in Trouble.

A Strong, Wise Personality—Pleasant to the Sight—her very Step Decisive and Firm—with a keen sense of Humour. Her merry Laugh and Cheerful Spirit at the Supper Table taking some of the Tiredness out of the long day's work.

A well-educated Woman—managing and leading the Chapel Choir with a Musical, well-modulated Voice. She tolerated no slouching, lazy attitude at Practice—"At Attention everyone."

She gave more than she asked. One of those rarely found, Strong, Valuable Pillars of the Profession, on which so much can be grafted. She never descended to discuss the Authorities or Management of the Hospital with Equals, much less Subordinates.

She perhaps gained more deep Respect and Admiration than Love, but she set a fine Copy, and pulled many weaker Souls to greater Heights by her consistent determination to be First Rate if possible.

A Memory to dwell upon with Gratitude for the privilege of knowing so brave a Leader. Well fitted for the post of Home Sister, it is doubtful, if she could or would have succeeded as "Matron." She was no Politician, and somewhat intolerant of Men or Women she disapproved of.

Abstemious to a fault, she had contempt for self-indulgence and weakness of will.

She lived McAndrew's Hymn—

"Now, a'together hear them lift their Lesson—theirs an' mine:
Law, Order, Duty an' Restraint, Obedience, Discipline."

She welcomed the "Royal National Pension Fund for Nurses" from the first. Her keen insight saw its value, and the opportunity it gave to teach Thrift.

"AN OLD SHOE."

### READERS ALL.

**[Every hospital in its day has had the privilege of being associated with workers who are or have been known to them, who constitute excellent types of the best side of British Nursing, and other types too. We want the readers of "The Hospital" to help the Editor to do justice to all these types by illustrating the circumstances and characteristics of, and the influence exercised by, individual workers within their knowledge. Five hundred words or less should suffice, but the facts, and the thoughts of the correspondent who sends them, will be welcome as material to be worked up. All contributions published will be paid for. There are some splendid sisters up and down the country, we remember with gratitude and hope.—Ed. The Hospital.]**

☛ The previous article appeared in "The Hospital" of October 27, 1917.

